# Mapping Kansas City cardiomyopathy, Seattle Angina, and minnesota living with heart failure to the MacNew-7D in patients with heart disease

**DOI:** 10.1007/s11136-024-03676-2

**Published:** 2024-06-05

**Authors:** Sameera Senanayake, Rithika Uchil, Pakhi Sharma, William Parsonage, Sanjeewa Kularatna

**Affiliations:** 1 Health Services and Systems Research, Duke-NUS Medical School, Singapore, Singapore; 2 Australian Centre for Health Services Innovation and Centre for Healthcare Transformation, School of Public health and Social Work, Faculty of Health, Queensland University of Technology, Brisbane, QLD 4059 Australia; 3 National Heart Research Institute Singapore, National Heart Centre Singapore, Singapore, Singapore; 4 Royal Brisbane and Women’s Hospital, Metro North Health, Brisbane, QLD Australia

**Keywords:** Mapping, Cross walk, Utility, MacNew-7D, Preference-based instruments

## Abstract

**Introduction:**

The Kansas City Cardiomyopathy Questionnaire (KCCQ), Seattle Angina Questionnaire (SAQ), and Minnesota Living with Heart Failure Questionnaire (MLHFQ) are widely used non-preference-based instruments that measure health-related quality of life (QOL) in people with heart disease. However, currently it is not possible to estimate quality-adjusted life-years (QALYs) for economic evaluation using these instruments as the summary scores produced are not preference-based. The MacNew-7D is a heart disease-specific preference-based instrument. This study provides different mapping algorithms for allocating utility scores to KCCQ, MLHFQ, and SAQ from MacNew-7D to calculate QALYs for economic evaluations.

**Methods:**

The study included 493 participants with heart failure or angina who completed the KCCQ, MLHFQ, SAQ, and MacNew-7D questionnaires. Regression techniques, namely, Gamma Generalized Linear Model (GLM), Bayesian GLM, Linear regression with stepwise selection and Random Forest were used to develop direct mapping algorithms. Cross-validation was employed due to the absence of an external validation dataset. The study followed the Mapping onto Preference-based measures reporting Standards checklist.

**Results:**

The best models to predict MacNew-7D utility scores were determined using KCCQ, MLHFQ, and SAQ item and domain scores. Random Forest performed well for item scores for all questionnaires and domain score for KCCQ, while Bayesian GLM and Linear Regression were best for MLHFQ and SAQ domain scores. However, models tended to over-predict severe health states.

**Conclusion:**

The three cardiac-specific non-preference-based QOL instruments can be mapped onto MacNew-7D utilities with good predictive accuracy using both direct response mapping techniques. The reported mapping algorithms may facilitate estimation of health utility for economic evaluations that have used these QOL instruments.

**Supplementary Information:**

The online version contains supplementary material available at 10.1007/s11136-024-03676-2.

## Introduction

Economic evaluations that use Quality Adjusted Life Years (QALYs) as the measure of effectiveness, are often used to inform decisions regarding the efficient allocation of limited healthcare resources [1]. QALYs combine the years of life gained due to an intervention with the health-related quality of life (HRQoL) of those years gained, into a single index [2]. QALYs are calculated using the formula – years of life (survival) x utility value (quality of life) [3]. Preference-based instruments are used to calculate the utility component of QALY, and contain two main components: (a) a multi-attribute descriptive system that includes a set of ‘question and response’ categories that attempt to describe an individual’s health and (b) the utility formula or algorithm that converts the responses into an index of utility on a 0.00 (death) and 1.00 (perfect health state) scale [4].

Disease-specific measures of quality of life are more sensitive to the symptoms of a particular disease [5]. For example, a disease-specific questionnaire for heart disease will measure the severity of symptoms related to heart disease more accurately than a generic measure [6, 7]. However, most disease-specific quality-of-life instruments are non-preference-based and cannot directly generate utility estimates. Non-preference-based instruments are characterized by their ability to measure, but not value, health states, which limits their use in economic evaluations. Until recently, there were no heart disease-specific preference-based instruments; thus, generic instruments such as the EQ-5D were widely used [8]. To address the need for a heart disease-specific preference-based measure, we developed a new heart disease-specific classification system, MacNew-7D, in 2022 [9, 10].

Heart diseases are considered to be one of the leading causes of morbidity and mortality worldwide. Heart diseases are responsible for an estimated 17.9 million deaths each year, accounting for approximately 31% of all deaths globally, underscoring their status as a leading cause of mortality [11]. Advancements in science and technology have led to a surge in heart disease related interventions. Most of these interventions, however, come with a high cost, posing a significant challenge as resources are limited. Consequently, individual interventions need to be evaluated for their value for money in an effort to improve health system efficiency. CUA related to heart disease can be strengthened by using disease-specific preference-based instruments, such as the MacNew-7D. Utilising such instruments significantly increases the accuracy of the evaluations. Interventions that have used cardiac disease-specific, non-preference-based instruments need to convert non-preference-based scores to preference-based utility scores, which can be used in CUA. To achieve this, mapping algorithms can be deployed.

Mapping is a way of allocating utility values from preference-based instruments to disease-specific non-preference based instruments [12]. Many disease-specific non-preference-based quality of life instruments, including those related to heart disease, have been successfully mapped using mapping algorithms [13, 14]. The aim of this study was to create mapping algorithms that would allow scores of Kansas City Cardiomyopathy Questionnaire (KCCQ), Seattle Angina Questionnaire (SAQ), and Minnesota Living with Heart Failure Questionnaire (MLHF) to be translated into utility values that could be applied to cost utility studies of people with heart disease. The study will provide utility values for these quality-of-life instruments that can be used to calculate QALYs and facilitate cost utility analysis.

## Methods

### Study population

The analysis included data of 493 participants (180 with heart failure and 313 with angina) with documented evidence of heart failure and stable or unstable angina, recruited from cardiology out-patient clinics at Royal Brisbane and Women’s Hospital (RBWH) hospital between January 2018 and March 2018.

After obtaining written informed consent, study participants completed a three-sectioned questionnaire. The first section included the participant’s socio-demographic information such as age, sex, and diagnosis. The second section included their responses for MLHF, KCCQ, and SAQ. The third section included their response for MacNew Heart Disease health-related quality of life instrument (MacNew). Institutional ethics committee approval was obtained from the Griffith University Human Research Ethics Committee (reference no. 2117/069).

### Instruments

The source instrument for mapping KCCQ, MLHF, and SAQ and the target instrument was MacNew-7D.

#### Kansas City cardiomyopathy questionnaire

The KCCQ is a self-administered questionnaire developed to create a valid and disease-specific health status measure for patients suffering from heart failure [15]. The KCCQ consists of 7 domains with 12 items measured on Likert scales with 5–7 response options. The five domains quantified are physical limitation, symptom frequency, symptom severity, symptom burden, social limitations, self-efficacy, and quality of life. Scores are scaled from 0 to 100 with higher scores indicating better HRQoL [16].

#### Minnesota living with heart failure questionnaire

The MLHF Questionnaire is most commonly used for patients with heart failure, to quantify HRQoL [17] It includes two domains, physical and emotional, consisting of 21 items which are rated on a six point Likert scale [17]. The total score (or sum score) ranges from 0 to 105, with higher scores indicating worse HRQoL [17].

#### Seattle angina questionnaire

The SAQ is one of the most commonly used measures of disease-specific health status in patients with angina [18]. The questionnaire includes 5 domains with 19 items, quantifying physical limitation scale, angina stability scale, angina frequency scale, treatment satisfaction scale, and quality of life scale. Each domain can be scored between 0 and 100, with higher scores indicating better quality of life [18].

#### MacNew-7D questionnaire

Kularatna et al. used the original 27 itemed MacNew-7D questionnaire to develop the heart disease specific preference-based instrument, MacNew-7D [10]. The MacNew-7D classification system has seven dimensions with four levels in each: physical restriction; excluded from doing things with other people; worn out or low in energy; frustrated, impatient or angry; unsure and lacking in self-confidence; shortness of breath; and chest pain. These seven dimensions with four levels allow 16,284 (4 [7]) possible health states to be defined and the utility value set range from − 0.4456 (minus value indicating worse than death) to 1.000 (perfect health) for health states defined by the classification system [10].

### Statistical analysis

Socio-demographic characteristics were summarised using mean (standard deviation [SD]) and median (interquartile range [IQR]) for continuous variables and frequency (percentage) was used for categorical variables. Spearman’s correlation was used to describe the correlation between the KCCQ, MLHF, and SAQ scores and the MacNew-7D utility scores. Guildford’s criteria was used to interpret the magnitude of correlation coefficient [19]. As per the criteria, the correlation coefficient is divided into five categories on the bases of their strength of association. These categories are as follows; very low (r: 0.00–0.20), low (r: 0.21–0.40), moderate (r: 0.41–0.60), high (r: 0.61–0.80) and very high (r: 0.81–1.00).

Direct response mapping was used in this study. Currently, there is not one specific regression method that is considered to be the best predictive model that fits all data sets [19]. In order to compensate for this uncertainty, four regression techniques were used on the same data set during direct mapping and the best method was decided on the basis of validation parameters. The four methods used were Gamma GLM, Bayesian GLM, Linear regression with stepwise selection and Random Forest. Each of the four techniques has the capacity to cope with ceiling effect, heteroscedasticity, skewness and/or the potential presence of outlier [19].

Regression techniques were used to develop the direct mapping algorithm. Six sets of independent variables were considered to predict the MacNew-7D utility score: prediction using KCCQ, MLHFQ and SAQ domain scores (*n* = 3) and total scores (*n* = 3).

On the basis of previous literature, squared terms of item scores and domain scores were added as independent variable in order to account for the non-linear relationship between MacNew-7D utility scores and KCCQ, MLHFQ, and SAQ score [19]. Age was included in order to improve the predictive performance.

Model1

$$\eqalign{MacNew\,7D\,utility\,score &= {\beta}_{0}+ \sum _{j=1}^{m}{\beta }_{j}* \left(\begin{array}{c}KCCQ\\ MLHFQ\\ SAQ\end{array}\right)\text{i}\text{t}\text{e}\text{m} \text{s}\text{c}\text{o}\text{r}\text{e}\cr &\quad+\sum _{j=1}^{m}{\beta }_{j}* \left(\begin{array}{c}KCCQ\\ MLHFQ\\ SAQ\end{array}\right){\text{i}\text{t}\text{e}\text{m} \text{s}\text{c}\text{o}\text{r}\text{e}}^{2}\cr &\quad+ {\beta }3\text{*}\text{A}\text{g}\text{e}}$$

Model2

$$\eqalign{MacNew\,7D\,utility\,score &= {\beta}_{0}+ \sum _{j=1}^{m}{\beta }_{j}* \left(\begin{array}{c}KCCQ\\ MLHFQ\\ SAQ\end{array}\right)\text{d}\text{o}\text{m}\text{a}\text{i}\text{n} \text{s}\text{c}\text{o}\text{r}\text{e}\cr &\quad+\sum _{j=1}^{m}{\beta }_{j}* \left(\begin{array}{c}KCCQ\\ MLHFQ\\ SAQ\end{array}\right){\text{d}\text{o}\text{m}\text{a}\text{i}\text{n} \text{s}\text{c}\text{o}\text{r}\text{e}}^{2}\cr &\quad+ {\beta }3\text{*}\text{A}\text{g}\text{e} }$$

### Assessing regression model performance

Goodness of fit of the models was assessed using root mean square (RMSD), R-squared value, and mean absolute error (MAE). RMSD is calculated as the root square value of the mean squared differences between the actual and predicted MacNew-7D [19]. It is the sum of variance and squared bias, where bias is representative of the difference between the population’s true value and the predictive value [20]. The mean absolute error is calculated as the mean of the absolute differences between the actual and predicted MacNew-7D scores [19]. Greater preference was put on MAE performance as it is less sensitive to outliers and easy to interpret [19]. Furthermore, AIC (Akaike Information Criterion) and BIC (Bayesian Information Criterion) were also used for model selection, where a lower value suggests a better-fitting model.

Due to the absence of an external validation dataset, we employed a three-fold cross-validation strategy to evaluate the performance of our regression models, using the ‘trainControl’ function from the ‘caret’ package in R. In three-fold cross-validation, the entire dataset is divided into three equal subsets or “folds”. The model is then trained on two of these folds (or two-thirds of the data) and validated, or tested, on the remaining third, and this process is repeated three times so that each fold serves as the validation set once. The performance indicators of the model (RMSD, R-squared value, and MAE) were then averaged over the three folds to obtain a more robust and generalized measure of the model’s predictive performance. The “Mapping onto Preference-based measures reporting Standards (MAPS) checklist was followed in this study.

To comprehensively evaluate the performance of our mapping algorithms across various health states, we employed a simulation technique [21]. This method is particularly advantageous in situations where analysts need to use the models to simulate individual level MacNew-7D data, rather than generate expected (e.g., mean cohort) MacNew-7D values. To assess the model’s ability to accurately capture uncertainty, we compared the simulated MacNew-7D scores from our best-performing models with the original observed data. Such validation is essential in cost-effectiveness analysis, which often relies on long-term projections and simulations involving numerous hypothetical patients. To conduct the simulations, we incorporated both the patient-specific explanatory variables and the random error terms inherent to the statistical model. This approach ensures that our model’s predictions encompass both the systematic and random variations observed in real-world data. In order to demonstrate the model’s predictive accuracy and address uncertainty, we generated 1000 simulated data points for each observation in the three datasets (KCCQ, MLHFQ, and SAQ). Subsequently, we depicted the results by plotting the cumulative distribution functions (CDFs) for each dataset.

All statistical analyses were performed using R Software version 4.1.0.

## Results

### Sample characteristics

A total of 493 participants took part in the study and were divided into two samples. Sample one included patients diagnosed with heart failure (*n* = 180) and sample two included patients diagnosed with angina (*n* = 313) (Table 1). Sample one completed the KCCQ & MLHFQ and sample two completed the SAQ. The mean age of the study participants for sample one was 60.8 (SD 14.2) and more than half (68%) were males. The mean MacNew-7D utility was 0.726 (SD 0.196) and the median was 0.720 (IQR 0.622–0.899). For sample two, the mean age of the study participants was 64.5 (SD 10.5) and more than half (74.7%) were males. The mean MacNew-7D utility was 0.735 (SD 0.202) and median was 0.769 (IQR 0.594–0.921).

**Table 1:** Patient characteristics

Characteristic	Sample 1 (*n* = 180) (KCCQ & MLHFQ)	Sample 2 (*n* = 313) (SAQ)
*Age (years)*		
Mean (SD)	60.8 (14.2)	64.5 (10.5)
Median (IQR)	62.0 (52.0–70.2)	64.8 (58.6–73.0)
*Sex*		
Male (%)	123 (68.3)	234 (74.7)
Female (%)	57 (31.7)	79 (25.3)
*Mac New 7D utility*		
Mean (SD)	0.726 (0.196)	0.735 (0.202)
Median (IQR)	0.720 (0.622–0.899)	0.769 (0.594– 0.921)
*Kansas City Cardiomyopathy Questionnaire (Max score 100)*		
Physical Limitation - Mean (SD)	67.7 (27.2)	
Symptom Stability - Mean (SD)	53.4 (17.8)	
Symptom Frequency - Mean (SD)	49.6 (26.3)	
Symptom Burden - Mean (SD)	78.7 (22.4)	
Self-Efficacy - Mean (SD)	80.6 (22.3)	
Quality of Life - Mean (SD)	72.1 (25.7)	
Social Limitation - Mean (SD)	67.3 (29.2)	
*Minnesota Living with Heart Failure*		
Physical dimension score (max 40) - Mean (SD)	27.4 (10.2)	
Emotional dimension score (max 25) - Mean (SD)	19.0 (6.7)	
*Seattle angina questionnaire*		
Physical Limitation scale		70.2 (25.5)
Angina stability scale		52.3 (19.7)
Angina frequency scale		86.7 (20.4)
Treatment satisfaction scale		86.8 (17.6)
Quality of life scale		70.2 (25.5)

KCCQ = Kansas City Cardiomyopathy Questionnaire, MLHFQ = Minnesota Living with Heart Failure Questionnaire, SAQ = Seattle Angina Questionnaire, SD = Standard Deviation, IQR = Interquartile Range

The study showed a strong correlation (*p* < 0.001) between the MacNew-7D and several domains across the KCCQ, MLHFQ, and SAQ. For KCCQ, this included physical limitation (*r* = 0.62), symptom frequency (*r* = 0.58), symptom burden (*r* = 0.67), quality of life (*r* = 0.68), and social limitation (*r* = 0.65). A moderate correlation was found with self-efficacy (*r* = 0.34), while a weak correlation was found with symptom stability (*r* = 0.09). A highly strong correlation was observed between MacNew-7D and the two domain scores of MLHFQ (*r* = 0.82 and *r* = 0.78). A strong correlation (*p* < 0.001) was observed between MacNew-7D scores and the physical limitation scale (*r* = 0.66), angina frequency scale (*r* = 0.54), and quality of life scale (*r* = 0.59) domains of the SAQ. Treatment satisfaction scale (*r* = 0.39) showed moderate correlation (Table 2).

**Table 2:** Correlation coefficients of MacNew-7D and the domains of the three heart disease-specific quality of life instruments

Instrument and domains	Spearman correlation	*P*-value
*Kansas City Cardiomyopathy Questionnaire*		
Physical Limitation	0.62	< 0.001
Symptom Stability	0.09	0.2122
Symptom Frequency	0.58	< 0.001
Symptom Burden	0.67	< 0.001
Self-Efficacy	0.34	< 0.001
Quality of Life	0.68	< 0.001
Social Limitation	0.65	< 0.001
*Minnesota Living with Heart Failure*		
Physical dimension score	0.82	< 0.001
Emotional dimension score	0.78	< 0.001
*Seattle angina questionnaire*		
Physical Limitation scale	0.66	< 0.001
Angina stability scale	0.06	0.2364
Angina frequency scale	0.54	< 0.001
Treatment satisfaction scale	0.39	< 0.001
Quality of life scale	0.59	< 0.001

### Validation

In the absence of an external validation dataset, predictive performance of the models was assessed using a three-fold cross validation method. All models were assessed for goodness of fit using the RMSD, R-squared and MAE. The best models to predict the MacNew-7D utility scores using the KCCQ, MLFHQ, and SAQ item scores and domain scores were selected based on their performance in the cross-validation step, with more weight put on the MAE following evidence in the literature.

Adding squared terms of both the items and the domain scores to the GLM and Bayesian GLM, led to no improvement in the MAE. Therefore, these squared terms were excluded from the final model of these two regression models. However, the squared terms were retained in the other two models (Linear Regression and Random Forest) as the inclusion led to an improvement in the MAE. In the case of the Gamma GLM, Bayesian GLM, and Linear Regression, only those variables that were statistically significant were included in the final regression model. This strategy applied to both item and domain level predictions. In the Random Forest models, adding squared terms of the items and domains did result in an improvement in the MAE across all models, with the exception of the SAQ item level prediction model, where squared terms were excluded.

### Best performing models

For KCCQ prediction using item scores, the Random Forest model was observed to be the best method with the lowest MAE (0.0929), highest R-squared (0.5961) and the lowest RMSD (0.1272). Gamma GLM most accurately predicted minimum and maximum utility values (difference of 0.0020 & 0.0491 to observed values). In MLHFQ, Random Forest had the lowest MAE (0.0818), while Bayesian GLM had the highest R-squared (0.7001) and lowest RMSD (0.1090). Gamma GLM predicted most accurately minimum and maximum utility values (difference of 0.0025 & 0.0657 to observed values). In the SAQ, Random Forest had the lowest MAE (0.0993), highest R-squared (0.5617), and lowest RMSD (0.1346). Gamma GLM predicted most accurately the minimum utility value (difference of 0.0496 to observed value) and Linear regression with stepwise selection predicted most accurately the maximum utility value (difference of 0.0657 to observed value). Based on MAE performance, the Random Forest method is observed to be the best model for predicting utility value using item scores for KCCQ, MLHF, & SAQ. However, it tended to over-predict severe health states. For example, the predicted minimum utility value for KCCQ, MLHF, & SAQ had a difference of 0.17979, 0.2772, & 0.1655 respectively to the observed values.

For KCCQ prediction using domain scores, Random Forest was observed to be the best method with the lowest MAE (0.0939). Gamma GLM predicted minimum & maximum utility value (difference of 0.1736 and 0.0131 to observed value) most accurately. In MLHF, Bayesian GLM had the lowest MAE (0.0828), highest R-squared (0.6928), and lowest RMSD (0.1097). Random Forest predicted most accurately minimum & maximum utility value (difference of 0.2331 & 0.056 to observed value). In SAQ, Linear regression with stepwise selection had the lowest MAE (0.0995), highest R-squared (0.5396), and lowest RMSD (0.1377) and also predicted most accurately the maximum utility value (difference of 0.323 to observed value). Bayesian GLM most accurately predicted the minimum utility value (difference of 0.1926 to observed value). Based on MAE performance, the random forest method was found to be the best model for predicting utility values using domain scores for KCCQ. Similar to item score, it tended to over-predict severe health states (difference of 0.2364 to observed value). For MLHF and SAQ, the Bayesian GLM was the best model, however, it under predicted severe health states (difference of 0.2543 and 0.1926 respectively).

AIC and BIC values are outlined in supplementary Table 1. These criteria were not applicable to the Random Forest models due to the nature of this non-parametric approach. The analysis revealed that for domain level predictions, the MLHFQ Bayesian GLM and the SAQ Linear Regression with stepwise selection demonstrated the lowest AIC and BIC values, indicating their superior fit among the evaluated models, consistent with other performance indicators.

**Fig. 1:**
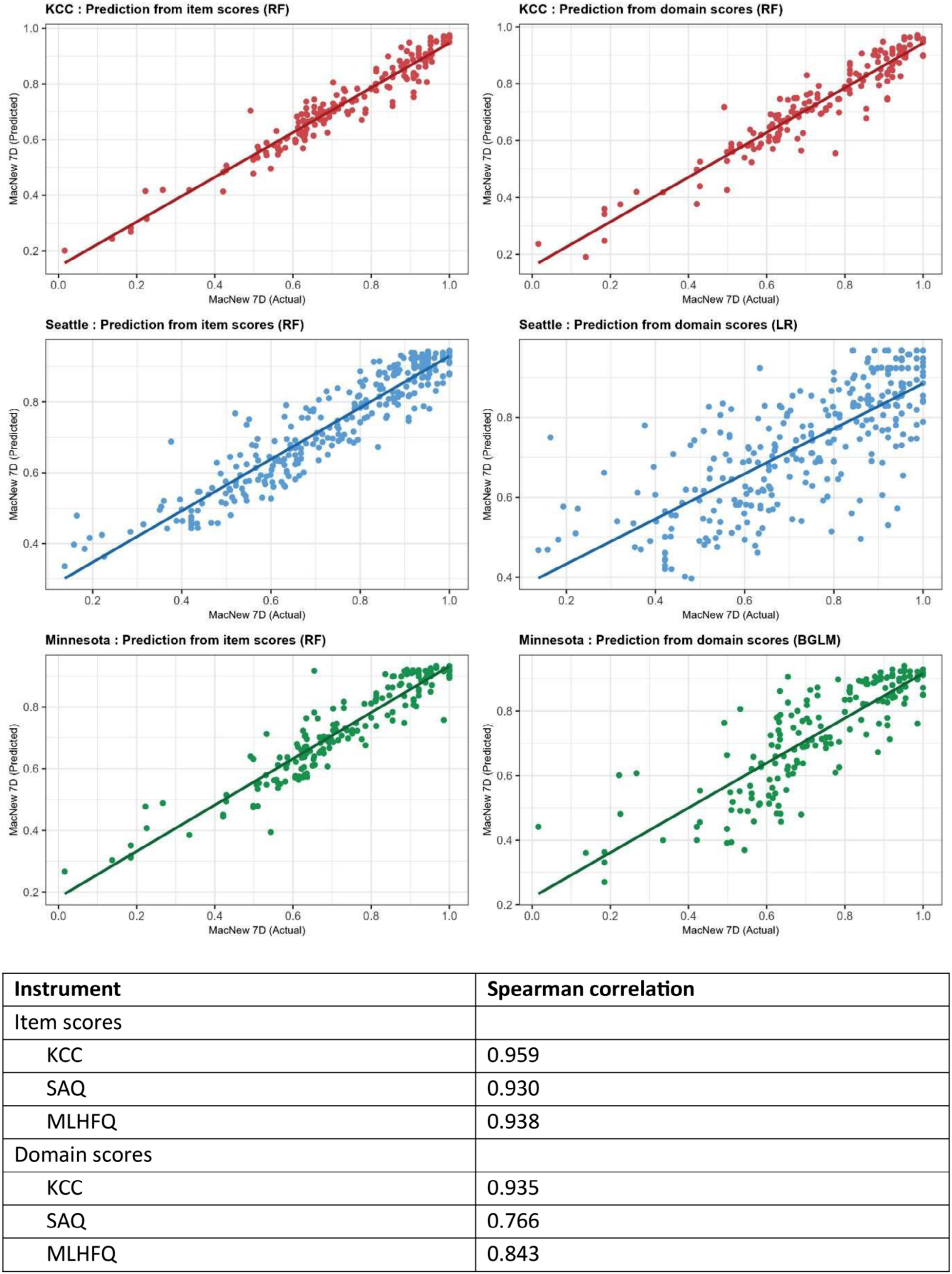
Scatter plot of observed versus predicted Mac New 7D. Line of equality between observed and predicted values (solid line). RF = Random Forest; BGLM = Bayesian GLM; LR = Linear Regression

**Fig. 2:**
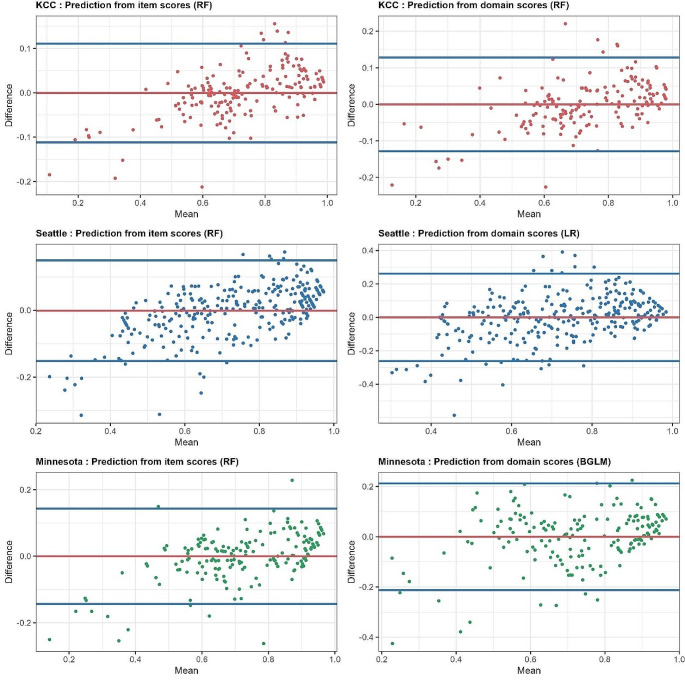
Bland and Altman plot of differences between the actual and the predicted Mac New 7D utility scores. RF = Random Forest; BGLM = Bayesian GLM; LR = Linear Regression

Figure 1 illustrates the scatter plots of actual vs. predicted MacNew-7D using the selected best performing models. A strong linear positive correlation is observed between the actual and predicted MacNew-7D scores when using item scores for KCC, SAQ, & MLHF as well as for KCC & MLHF when using domain scores, with majority data points lying close to the line of best fit. However, when predicting MacNew-7D values using domain scores for SAQ, a moderately strong positive correlation (*r* = 0.766) with data points scattered away from the line of best fit are observed. The Bland-Altman plot in Fig. 2 shows a vast majority of data points lying close to the mean difference and within the limits of agreement, indicating good agreement between the actual and the predicted MacNew-7D values.

**Fig. 3:**
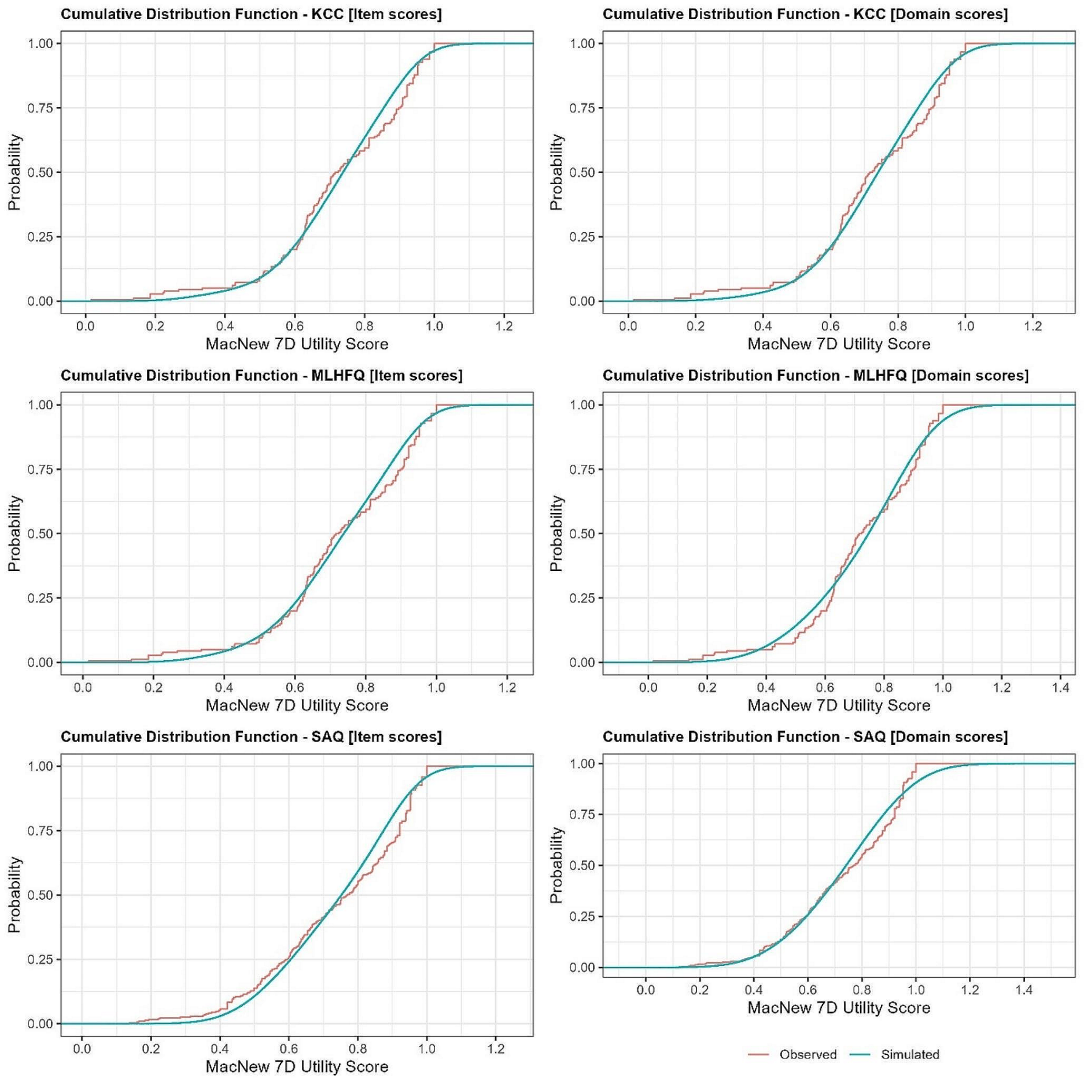
Cumulative distribution function of observed and simulated MacNew-7D values of the best performing models

The cumulative distribution function plots (Fig. 3) exhibit a strong level of agreement between the observed and simulated MacNew 7D utility scores across the three datasets, KCC, MLHFQ, and SAQ. Both the item and domain scores demonstrate that the simulation model accurately captures the distribution of the actual data, indicating a precise fit. This is particularly evident as the cumulative distribution function curves for the simulated data closely mimic those of the observed data across the entire range of utility scores. It is worth noting that in the MLHFQ and SAQ domain scores, there is a slight deviation near the upper limit, which suggests potential differences at extreme values. Overall, the consistency of these plots across different instruments emphasises the reliability of our simulation model in reflecting real-world patient-reported outcomes.

Online resource files contain R scripts and detailed descriptions which can be used to predict the MacNew-7D utility scores from the best prediction models (Table 3).

**Table 3:** Goodness of fit results from three-fold cross-validation

Instrument	Method	Mean utility	Min utility	Max utility	RMSD	*R*-squared	MAE
**Prediction using item scores**							
KCCQ (*n* = 180)	Observed	0.7264	0.0159	1.0000			
	Gamma GLM (link = identity)	0.7408	0.0179	1.0491	0.1498	0.5237	0.1148
	Bayesian GLM	0.7264	0.2720	0.9509	0.1323	0.5573	0.0991
	Linear regression with stepwise selection	0.7264	0.2625	0.9569	0.1459	0.4798	0.1091
	**Random forest**	0.7274	0.1956	0.9800	0.1272	0.5961	**0.0929**
MLHFQ (*n* = 180)	Observed	0.7264	0.0159	1.0000			
	Gamma GLM (link = identity)	0.7378	0.0184	1.0657	0.1299	0.6082	0.0990
	Bayesian GLM	0.7264	0.2741	0.9256	0.1090	0.7001	0.0861
	Linear regression with stepwise selection	0.7264	0.2512	0.9513	0.1414	0.5480	0.1058
	**Random forest**	0.7265	0.2931	0.9334	0.1144	0.6686	**0.0818**
SAQ (*n* = 317)	Observed	0.7351	0.1373	1.0000			
	Gamma GLM (link = identity)	0.7353	0.1869	0.9681	0.1455	0.4924	0.1108
	Bayesian GLM	0.7351	0.2599	0.9710	0.1435	0.4993	0.1080
	Linear regression with stepwise selection	0.7351	0.3425	1.0131	0.1610	0.4130	0.1163
	**Random forest**	0.7357	0.3524	0.9431	0.1346	0.5617	**0.0993**
**Prediction using domain scores**							
KCCQ (*n* = 174)	Observed	0.7314	0.0159	1.0000			
	Gamma GLM (link = identity)	0.7364	0.1895	1.0567	0.1538	0.5192	0.1168
	Bayesian GLM	0.7314	0.2468	0.9522	0.1245	0.6003	0.0955
	Linear regression with stepwise selection	0.7314	0.2757	0.9673	0.1373	0.5298	0.1024
	**Random forest**	0.7317	0.2523	0.9498	0.1275	0.5799	**0.0939**
MLHFQ (*n* = 180)	Observed	0.7264	0.0159	1.0000			
	Gamma GLM (link = identity)	NA	NA	NA	NA	NA	NA
	**Bayesian GLM**	0.7264	0.2702	0.9393	0.1097	0.6928	**0.0828**
	Linear regression with stepwise selection	0.7264	0.2702	0.9393	0.1137	0.6675	0.0855
	Random forest	0.7269	0.2490	0.9440	0.1149	0.6691	0.0834
SAQ (*n* = 313)	Observed	0.7355	0.1373	1.0000			
	Gamma GLM (link = identity)	0.7351	0.3467	0.9380	0.1379	0.5391	0.1012
	Bayesian GLM	0.7355	0.3299	0.9431	0.1403	0.5199	0.1024
	**Linear regression with stepwise selection**	0.7355	0.3970	0.9677	0.1377	0.5396	**0.0995**
	Random forest	0.7354	0.3598	0.9450	0.1397	0.5278	0.1027

KCCQ = Kansas City Cardiomyopathy Questionnaire, MLHFQ = Minnesota Living with Heart Failure Questionnaire, SAQ = Seattle Angina Questionnaire, RMSD = Root Mean Square Deviation, MAE = Mean Absolute Error, GLM = Generalised Linear Model, NA = Not Applicable. *Bold = Best performing models in KCCQ, MLHFQ, and SAQ

## Discussion

This study aimed to map three commonly used cardiac disease health-related quality of life questionnaires to the MacNew-7D utility values in patients with heart disease. The results provide insights into the relationships between these instruments and the utility values derived from the MacNew-7D. Due to the lack of comparable mapping studies between KCCQ, MLHF, & SAQ to MacNew-7D it is difficult to perform a direct comparison of validity parameters of the study to current literature. This mapping facilitates estimation of utility values from any of the heart disease specific quality of life instruments used in this analysis.

The correlation analysis demonstrated significant associations between MacNew-7D and the various domains of the questionnaires. Moderate correlations were observed between MacNew-7D and all domains of KCCQ, MLHF, and SAQ except the symptom stability domain of KCCQ. A moderate correlation indicates that the MacNew-7D is valid and is capturing aspects of health-related quality of life that are relevant to the specific domains of these questionnaires. This may be important for clinicians and researchers who use these instruments to assess the impact of interventions or disease progression on patients’ health-related quality of life [22–24]. Moreover, it may indicate valuable information about the effectiveness of treatments or interventions targeted at improving the specific domains of interest. Previously, policy makers and researchers have utilised mapping results to estimate utility scores, QALYs, and consequently incremental cost effectiveness ratios (ICERs), and incorporated them into decision-making processes in different research fields, for example, dialysis treatment [25], epilepsy management [26], and joint replacement surgeries [27]. Our study may facilitate economic evaluations, for example QALYs and cost-utility analyses in heart disease. This may be used for decision-making related to resource allocation, reimbursement decisions, health policy formulation, and treatment guidelines in the field of cardiovascular diseases. The MacNew-7D may instigate a more precise and specific assessment of HRQoL in individuals with heart disease, that may further play a key role in delivering informed and targeted healthcare decisions.

Due to the absence of an external validation dataset, a three-fold cross-validation method was employed to validate the mapping models. Cross-validation is a prevalent method used in machine learning when there is limited dataset or when an external validation dataset is not available [28]. It involves splitting the dataset into multiple subsets [28]. By using cross-validation, an analyst can estimate how well the mapping models generalize to unfamiliar data within the same dataset [29]. In this case, it assessed the model’s ability to obtain the relationships between the predictors (e.g., KCCQ, MLHFQ, SAQ) and the target variable (MacNew-7D utility scores) and indicated the model’s performance on new data from the similar populations. Cross-validation is a broadly used method in various fields including cancer studies, dermatology, and orthopaedics [30–32]. In these studies, performance of the models was assessed using RMSD, R-squared, and MAE. Therefore, cross-validation provides a rigorous approach to assess the performance and generalisability of mapping models in the absence of an external validation dataset. In our analysis, we observed that the R-squared, RMSE, and MAE values were higher compared to some mapping studies, including the study by Klapproth et al. [33]. This study, which developed optimal models for mapping the EQ-5D-5 L crosswalk from the PROMIS-29 in the UK, France, and Germany, reported lower values of these metrics. Specifically, their nRMSE values were 0.076, 0.075, and 0.079 for the UK, France, and Germany, respectively, which are lower than those observed in our study. The differences in these metrics between our study and studies like Klapproth et al. [33] could be attributed to various factors, including the nature of the health conditions assessed, the specific patient populations involved, and the methodological approaches employed in model development. These factors underscore the complexity and variability inherent in mapping studies and highlight the need for context-specific considerations in the interpretation and application of such models.

For the KCCQ and SAQ, the Random Forest model exhibited the best performance. In general, the Random Forest model has been found to outperform other models in several studies in different healthcare contexts [34–36]. The Random Forest algorithm’s ability to handle complex interactions, high-dimensional data, and reduce overfitting makes it a popular choice in machine learning-based healthcare research [37, 38]. However, it is important to note that the choice of the best-performing model may vary depending on the specific dataset and research question at hand [37]. The gamma GLM model accurately predicted the minimum and maximum utility values for KCCQ and SAQ. The gamma GLM model is particularly suitable for handling skewed and non-normally distributed data, which makes it a popular choice in mapping studies [39]. However, its performance in accurately estimating extreme values depends on the underlying distribution of the data and the assumptions of the model [39]. Other mapping studies also found that the gamma GLM model best predicted minimum and maximum utility value [40–42].

Similarly, for the MLHFQ, the Random Forest model achieved the lowest MAE, while the Bayesian GLM model had the highest R-squared and lowest RMSD. The gamma GLM model accurately predicted extreme utility values. The Bayesian GLM model has performed well in comparatively few studies. One such study aimed to map the European Organisation for Research and Treatment of Cancer Quality of Life Questionnaire (EORTC QLQ-C30) onto the EuroQol Five-Dimensional Five-Level (EQ-5D-5 L) questionnaire in lung cancer patients. This study had highest R-squared and lowest RMSD compared to other mapping models tested [31]. The Bayesian approach in modelling allows for the incorporation of prior knowledge or beliefs about the parameters and uncertainties into the model [43]. Moreover, it provides a framework to estimate posterior distributions of the model parameters and to make probabilistic inferences about the relationships between variables [43]. It should be noted, however, that the random forest models tended to over-predict severe health states across all three questionnaires. This may imply that the predicted utility values for individuals with poorer health or more severe symptoms were higher than the observed values. In other words, the Random Forest models underestimated the impact of the disease on the quality of life for individuals with more severe cardiovascular conditions. This observation has practical implications in healthcare decision-making and resource allocation. Over-predicting severe health states could potentially lead to an underestimation of the burden of the disease and may affect the allocation of healthcare resources. However, over and under-predicting are common issues in mapping [22, 33, 44–46]. These may occur due to several reasons including when the model extrapolates beyond the observed data, when there is significant disparity in the distribution of different classes or categories, when the model fits the training data too closely, or if the training data has a limited representation of extreme cases [47, 48].

In comparing our study’s findings to existing mapping studies to EQ-5D, the most commonly used utility measure, we note several interesting parallels and distinctions. For instance, a study mapping the SAQ to EQ-5D in coronary health disease patients in China reported correlations (ranging from 0.62 to 0.71) similar to our findings. However, the smaller sample size in that study might limit comparability [49]. Another research mapping SAQ to EQ-5D observed weaker correlations (ranging from 0.18 to 0.59) compared to ours [50]. Regarding studies mapping the KCCQ to EQ-5D, they generally reported lower R-squared statistics (0.48 to 0.50) than what we found in our study [51]. Similarly, Hunger et al., in developing a mapping algorithm for Japanese and UK value sets, also reported lower R-squared statistics (0.45 to 0.52) [52]. This contrast may highlight the high sensitivity of MacNew-7D in assessing heart disease impacts. MacNew-7D, being specifically tailored to heart disease, including key dimensions like shortness of breath and chest pain [7], might capture nuances of heart disease effects more effectively than generic measures.

This suggests that for heart disease patients, a disease-specific instrument like MacNew-7D can provide a more accurate evaluation of HRQoL compared to generic utility measures. The initial concept of QALY was to enable comparison of cost-effectiveness across different conditions, leading to the prevalent use of generic preference-based measures. However, the recent trend towards condition-specific preference-based measures, such as MacNew-7D, has sparked a debate about the most effective approach for capturing patient experiences. Our MacNew-7D health state valuation study specifically highlights the substantial disutility linked to dimensions like shortness of breath and chest pain, aspects not as comprehensively captured by generic measures like EQ-5D. This suggests that while generic measures offer broad comparability, condition-specific measures can provide more detailed insights into certain patient experiences, especially when symptoms have significant clinical implications.

### Strengths and limitations

Including relatively large sample size and thorough validation process using cross-validation are few of the strengths of this study. The mapping models predicted MacNew-7D utility scores well, particularly item scores. Although MAE, RMSE, and R-squared are reliable parameters, these measures have limitations. It is recommended to consider multiple evaluation metrics and examine other aspects including the context and research question of the study, the interpretability of the model, and requirements of the analysis.

There are some limitations to consider. Firstly, the study sample only consisted of patients with heart failure and angina, which may limit the generalizability of the findings to other cardiovascular conditions. Additionally, the absence of an external validation dataset may introduce some uncertainty in the generalizability of the mapping models. Furthermore, the under-prediction of severe health states by the Random Forest models warrants further investigation and potential refinement of the mapping algorithms. Our study did not explore the use of mixture models due to the modest sample size and the complexity it would introduce. Recognizing this as a limitation, future studies with larger datasets may benefit from considering mixture models to potentially uncover latent sub-populations within heart disease patients, enhancing the precision of mapping algorithms.

## Conclusion

In conclusion, the mapping of the KCCQ, MLHFQ, and SAQ to the MacNew-7D in patients with heart disease showed promising results. The correlations between the questionnaires and the utility values derived from the MacNew-7D suggest that the MacNew-7D captures important aspects of health-related quality of life specific to cardiovascular disease. The mapping models demonstrated reliable predictive performance, although some limitations should be considered. Further research is needed to validate and refine the mapping algorithms and to explore their applicability to a wider range of cardiovascular conditions.

### Electronic supplementary material

Below is the link to the electronic supplementary material.

Supplementary Material 1

Supplementary Material 2

Supplementary Material 3

Supplementary Material 4
