# Supplementary material for: Mapping Kansas City cardiomyopathy, Seattle Angina, and minnesota living with heart failure to the MacNew-7D in patients with heart disease
Source: Qual Life Res. 2024 Jun 5;33(8):2151–63. doi: 10.1007/s11136-024-03676-2 (PMC11286692; doi:10.1007/s11136-024-03676-2)
Supplement: Supplementary file 1 — Supplementary Material 1 [file 11136_2024_3676_MOESM1_ESM.docx]

**Supplementary table 1:** AIC (Akaike Information Criterion) and BIC (Bayesian Information Criterion) values of the models used

| Prediction using item scores | | | |
| --- | --- | --- | --- |
| Instrument | Method | **AIC** | **BIC** |
| KCCQ (n=180) | Gamma GLM (link = identity) | -163.34 | -115.44 |
|  | Bayesian GLM | -223.66 | -198.12 |
|  | Linear regression with stepwise selection | -251.36 | -177.92 |
|  | Random forest | Not applicable | Not applicable |
|  |  |  |  |
| MLHFQ (n=180) | Gamma GLM (link = identity) | -181.05 | -149.13 |
|  | Bayesian GLM | -284.85 | -262.50 |
|  | Linear regression with stepwise selection | -297.36 | -233.50 |
|  | Random forest | Not applicable | Not applicable |
|  |  |  |  |
| SAQ (n=317) | Gamma GLM (link = identity) | -239.60 | -190.90 |
|  | Bayesian GLM | -339.30 | -298.09 |
|  | Linear regression with stepwise selection | -344.58 | -288.39 |
|  | Random forest | Not applicable | Not applicable |
|  | | | |
| Prediction using domain scores | | | |
| Instrument | Method | **AIC** | **BIC** |
| KCCQ (n=180) | Gamma GLM (link = identity) | -101.08 | -75.81 |
|  | Bayesian GLM | -230.74 | -205.47 |
|  | Linear regression with stepwise selection | -233.71 | -202.12 |
|  | Random forest | Not applicable | Not applicable |
|  |  |  |  |
| MLHFQ (n=180) | Gamma GLM (link = identity) | Not applicable | Not applicable |
|  | Bayesian GLM | -280.26 | -264.29 |
|  | Linear regression with stepwise selection | -280.26 | -264.29 |
|  | Random forest | Not applicable | Not applicable |
|  |  |  |  |
| SAQ (n=317) | Gamma GLM (link = identity) | -233.94 | -211.46 |
|  | Bayesian GLM | -347.45 | -324.97 |
|  | Linear regression with stepwise selection | -361.69 | -339.21 |
|  | Random forest | Not applicable | Not applicable |

KCCQ = Kansas City Cardiomyopathy Questionnaire, MLHFQ = Minnesota Living with Heart Failure Questionnaire, SAQ = Seattle Angina Questionnaire, RMSD = Root Mean Square Deviation, MAE = Mean Absolute Error, GLM = Generalised Linear Model, NA = Not Applicable.
